# OX130 Monoclonal Antibody Recognizes Human SIRPβ1 but Cross-Reacts on SIRPα from One Allele

**DOI:** 10.1089/mab.2015.0054

**Published:** 2016-02-01

**Authors:** Deborah Hatherley, Marie-Laure Aknin, Neil Barclay

**Affiliations:** Sir William Dunn School of Pathology, University of Oxford, Oxford, United Kingdom.

## Abstract

The SIRP family of myeloid-paired receptors are characterized by having both activating and inhibiting members with extracellular regions that are relatively similar. Making good reagents to these receptors is not straightforward, particularly as they are relatively polymorphic. We describe the production of a monoclonal antibody (MAb) called OX130 that recognizes both common alleles of the human activating SIRPβ1 receptor but also cross-reacts with one of the common alleles of the inhibitory human SIRPα receptor. Thus one might get different outcomes when this MAb is used in assays from different individuals and shows the importance of characterizing SIRP MAb in this way.

## Introduction

Paired receptors are groups of closely related membrane proteins found at the surface of NK cells and myeloid cells.^(1,2)^ They are characterized by containing at least one member that can give inhibitory signals; this is usually created by the recruitment of phosphatases through immunoreceptor tyrosine-based inhibition motifs (ITIM) in their cytoplasmic regions and at least one other member that can give activating signals through the association with an adapter such as DAP12 that contains immunoreceptor tyrosine-based activation motifs (ITAM) that can recruit kinases. The SIRP family consists of three members with three Ig-like domains: SIRPα gives inhibitory signals, SIRPβ1 gives activating signals, and SIRPγ probably does not signal.^(3)^ In addition there are some more distantly related proteins with different numbers of extracellular domains.^(4)^ Like many paired receptors, these proteins are diverging rapidly in evolution with most sequence differences in the N-terminal domain. There are two common alleles of SIRPα that differ markedly in sequence (13 differences in their N-terminal domains) and two SIRPβ1 (12 differences). Thus reagents that are specific for each class of proteins are not straightforward to produce. In this report, we describe the production of the OX130 MAb that recognizes both common alleles of SIRPβ1 but also binds one SIRPα1 allele.

## Material and Methods

### Production of SIRPβ1 MAb

Recombinant human SIRPβ1 protein comprising three extracellular immunoglobulin superfamily (IgSF) domains (termed SIRPβ1(1) with accession numbers NP_006056, residues 1-360) with a C-terminal STRH_6_ tag was expressed using the pEE14 vector in the CHO Lec3.2.8.1 cell line. Recombinant SIRPβ1(1) was purified by nickel affinity chromatography and used to immunize mice. Monoclonal antibodies were produced by standard procedures using the NS1 cell line. The MAbs were screened by ELISA on recombinant SIRP proteins, and those reactive with SIRP1 were cloned. One clone OX130 (mouse IgG) that also labeled SIRPβ1 cells by flow cytometry was characterized in detail by surface plasmon resonance analysis using a BIAcore™ 3000 (GE Healthcare, Little Chalfont, UK), employing previously described methods.^(5)^ For this analysis, the SIRP proteins were expressed as three domain constructs together with rat CD4d3 + 4 as an antigenic tag to enable binding to a rat CD4 MAb (OX68) that had been immobilized on the BIAcore CM5 chip (Table 1). The SIRP proteins tested were the SIRPα variants (1) and (10), as described previously,^(6)^ SIRPβ1 proteins (accession nos. NP_006056 and CAI21700), and SIRPγ (accession no. NP_061026), as described previously.^(7)^ Approximately 1000 response units (RU) of each recombinant SIRP protein were immobilized, including rat CD4 as a negative control protein. Human CD47 at 22 μM (prepared as described earlier^(5)^) was passed over the immobilized proteins, followed by OX130 MAb and by CD47 again to see if OX130 MAb binding prevented CD47 binding to SIRPα.

**Table 1.** OX130 MAb Blocks CD47 Binding to SIRPα(1)

*Protein immobilized*	*Level of immobilized protein*	*CD47 binding*	*CD47 binding post OX130 injection*
SIRPα(1)	862	154	22
SIRPα(10)	956	280	247
SIRPβ1(1)	1036	15	2

**FIG. 1.**
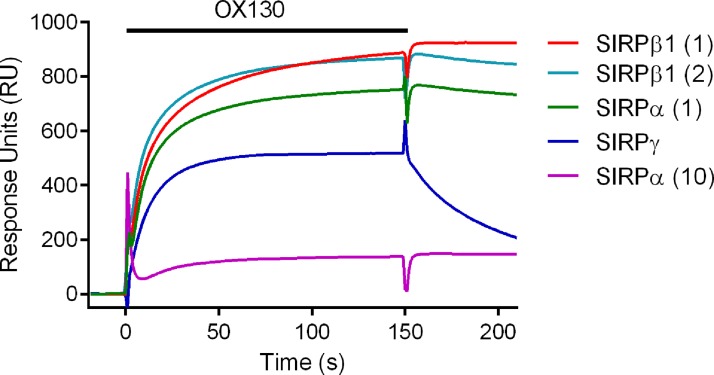
Analysis of OX130 MAb binding to SIRP proteins by SPR. Overlay of specific binding traces showing OX130 bound well to both alleles of SIRPβ1 but only one allele of SIRPα(1). There is some weak transient binding to SIRPγ. Immobilization levels of SIRP proteins shown are SIRPα(1) 862 response units (RU); SIRPα(10) 956 RU; SIRPβ1(1) 953 RU; SIRPβ1(2) 999 RU; and SIRPγ 1129 RU. Bar indicates where OX130 was passed over flow cell. Background signal determined on a control CD4 flow cell was subtracted from all traces. SPR, surface plasmon resonance.

BIAcore analysis showing response units (RU) of three SIRP proteins immobilized via their CD4 tag. RU values for CD47 passed over the proteins are shown. OX130 MAb was then passed over and CD47 passed over again. Specific binding responses were calculated by subtracting RU obtained for the immobilized control protein rat CD4 domains 3 and 4. The OX130 MAb gives almost complete inhibition of CD47 binding to SIRPα(1) but has no effect on SIRPα(10), to which it does not bind (see Fig. 1). SIRPβ1(1) shows minimal binding of CD47 as expected.

## Results and Discussion

### OX130 MAb recognizes human SIRPβ1

ELISA results showed that OX130 MAb bound recombinant SIRPβ1 (both alleles) but not SIRPα (only allele (10) was tested—see below) or SIRPγ (data not shown). In order to give a more quantitative comparison of the reactivity of OX130, the MAb was tested for binding using surface plasmon resonance (SPR) to various SIRP proteins that had been immobilized to a BIAcore chip through a CD4 tag with an overlay of the BIAcore traces (Fig. 1).

**FIG. 2.**
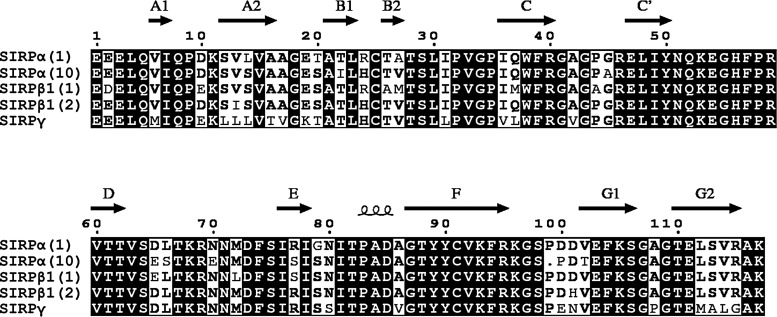
Sequence alignment of IgSF V-like domain of the SIRP-paired receptor family. Positions of the beta strands are represented by arrows as determined by the crystal structure of SIRPα(1). Identical residues are highlighted in black, and residues that are identical in at least three sequences are bold. Alignment was generated using ESPript 3.0 Web Server (http://espript.ibcp.fr).

The OX130 MAb recognized both common alleles of SIRPβ1 but not SIRPγ. However it reacted with one of the two common alleles of SIRPα but not the other. This binding prevented the subsequent binding of CD47, showing that the binding site of the MAb is in the N-terminal domain near the ligand binding domain.^(5)^ Examination of the amino acid sequences of the SIRP domain 1 illustrated the many differences between the SIRPα alleles and SIRPβ1 alleles (Fig. 2). One region that might be important in the specificity of OX130 is around residue 99 where the sequence is identical in the three SIRPs that bind OX130 but different in the other two SIRPs. Most SIRP reagents have not been tested on all these proteins so it is possible that other reagents may give different effects according to the alleles of SIRPα and SIRPβ1 present in an individual or indeed in a cell line.
